# Identification of Enterprise Financial Risk Based on Clustering Algorithm

**DOI:** 10.1155/2022/1086945

**Published:** 2022-04-12

**Authors:** Bingxiang Li, Rui Tao, Meng Li

**Affiliations:** School of Economics and Management, Xi'an University of Technology, Xi'an 710054, China

## Abstract

In order to solve the problem that corporate financial risks seriously affect the healthy development of enterprises, credit institutions, securities investors, and even the whole of China, the K-means clustering algorithm, the risk screening process, and the Gaussian mixture clustering algorithm, the risk screening process, are proposed; experiments have shown that although the number of high-risk companies selected by the K-means algorithm is small, only 9% of the full sample, the high-risk cluster can contain nearly 30% of the new “special treatment” companies. If the time period is extended to the next 5 years, this proportion will be higher. Finally we found that if the prediction of “special handling” events is used as the criterion for evaluating high-risk clusters, then K-means clustering can effectively screen out those risky companies that need to be treated with caution by investors. The validity of the experiment is verified.

## 1. Introduction

**Figure 1:**
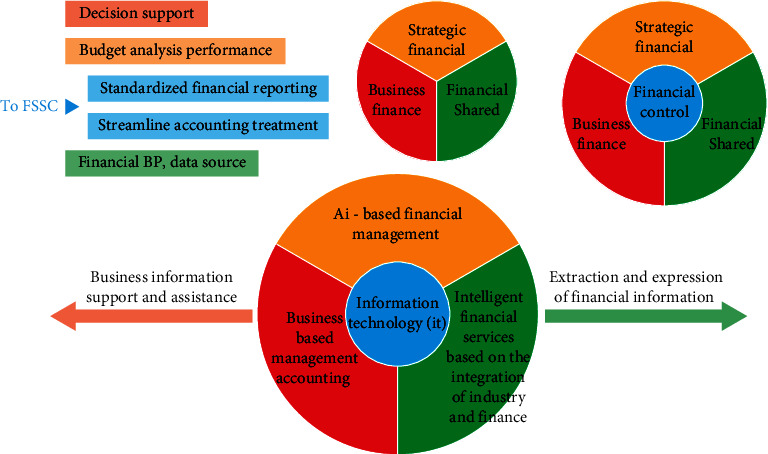
Identification of corporate financial risks.

The evaluation and control of corporate financial risk is an important research subject in the financial sector, as shown in Figure 1. Over the past four decades, financial risk has had a serious impact on corporate operations and financial market stability. In China, the problem of corporate finance risk has become more and more remarkable as the socialist market economy is established [1]. In a business environment, the industry faces a very difficult and ongoing relationship and business environment where businesses do not have access to all the information they need to do business. Trade: their markets face uncertainty, and the benefits of financial markets are there. As a result, businesses may benefit, suffer losses, or even lose money. China and the world: bankruptcies occur frequently every year due to corporate financial risks, many large conglomerates are in crisis, and it has had a major negative impact on the social and economic development of various countries [2]. For example, Yamashiro securities and Hokkaido Takushoku bank, and Babaihan International Group, got into trouble successively. Among the top 30 large-scale enterprise groups in South Korea, after Hanbao Group declared bankruptcy, a series of chaebol-level large-scale enterprise groups such as Sanmei, Danong, Jinro, Kia, Haitian, Newco, etc. also closed down or fell into business difficulties one after another; Eaton Business Group, Canada's largest commercial enterprise, also went bankrupt due to financial risk. China's Zheng Baiwen, Yinguangxia, Yi'an Technology, Dongfang Electronics, etc. are also faced with serious financial risks and even fall into financial crisis. Take the A-share listed companies (referred to as ST companies) that were specially dealt with in 2005 and 2006 as an example [3]. In 2005, 34 companies were specially dealt with, 31 companies were specially dealt with due to abnormal financial situation, and there are three companies specially addressed for other abnormal conditions. Of the 31 companies that were specially dealt with due to abnormal financial conditions, 26 companies were specially dealt with for two consecutive years of losses in 2003 and 2004 (including losses due to adjustments), 2 companies are because shareholders' equity is lower than the registered capital, that is, the net assets are lower than the face value and are specially treated, and the other 3 companies were specifically dealt with due to the CPA's opinion. In 2006, a total of 52 companies were specially dealt with, of these, 49 companies were given special treatment for two consecutive years of losses in 2004 and 2005. The remaining three companies were involved in major legal proceedings due to the opinions of certified public accountants, and the 2005 annual report was not published. The trading of the company's shares has been suspended for two consecutive months, and special treatment will be announced within the legal period. From this it can be seen that, in 2005 and 2006, in companies that were specially treated, the vast majority are facing serious financial risks. Unfortunately, so far, most Chinese companies have yet to incorporate financial risk management into their daily management; the corporate financial risk is not yet widely managed [4].

## 2. Literature Review

**Figure 2:**
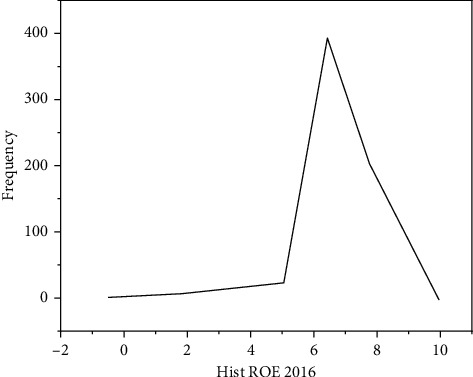
Histogram of return on equity of listed companies in 2016.

**Figure 3:**
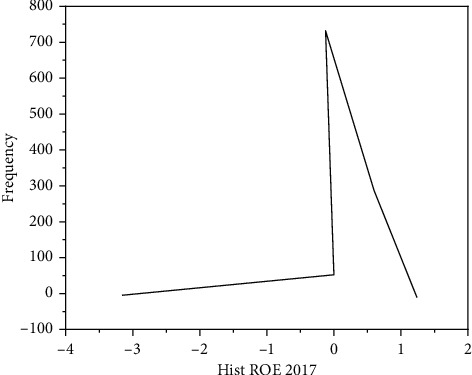
Histogram of return on equity of listed companies in 2017.

Most Chinese companies still need to incorporate financial risk management into their daily management, the corporate financial risk is not yet widely managed, which was put forward at the first insurance conference of the American Management Association [5]. Ilchuk and Shyshkina found that later, with the continuous development and improvement of risk management theory and practice, people began to gradually introduce these theories and practices into enterprise financial risk management [6]. Boiko and Gevrek found that, managing corporate financial risk, it is necessary to analyze the causes of enterprise financial risks and identify and measure financial risks; that is, the evaluation of enterprise financial risk is the basis; managing corporate financial risks, its purpose is to adopt economically reasonable control strategies and avoid or diversify risk, to avoid losses; that is, the control of enterprise financial risk is the key, as shown in Figures 2 and 3 [7]. Tereshchenko et al. argue that, however, judging from the current situation of Chinese enterprise practice and theoretical research, to effectively evaluate and control corporate financial risks in China, there are still big gaps. Under the planned economic system, the financial risks of Chinese enterprises are borne by the state; however, with development of socialist market economy and establishment of modern enterprise system with the further integration of China and the western enterprise system after China's entry into the WTO, enterprises should constantly establish financial risk awareness and possess the ability to undertake and resolve corporate financial risks [8]. Hemmer and Moore found that the external environment in which enterprises are located is complex and changeable, and this complex and changeable external environment will bring risks to enterprises [9]. The external environment faced by enterprises mainly refers to the macroeconomic environment, legal environment, market environment, social and cultural environment, resource environment, financial environment, etc. These external environmental factors exist for companies; for enterprises, it is difficult to predict accurately, and it cannot be changed, but it will have a great impact on the corporate financial risk. Ahmad et al. found that, for example, the rise in world crude oil prices has led to a rise in refined oil prices, it will increase the operating cost of the transportation company and reduce profits, and as a result, the expected revenue target cannot be achieved. In another example, enterprises fail to understand the government's macrocontrol intentions and did not act in accordance with the guidelines of the policy; this leads to the financial crisis of the enterprise [10]. Peng et al. believe that, in summary, if you cannot adapt to the complex and changing external environment, it will inevitably affect the normal production and operation activities of the enterprise, thereby causing operational and financial risks [11]. With economic globalization, the world economy has shown a trend of gradual integration, and the international competition among enterprises has become increasingly fierce, showing the “domestication of the international market, internationalization of the domestic market.” Gosain and Dahiya found that, in this context, only by formulating an appropriate internationalization strategy, reasonable allocation of resources is done on a global scale, in order to gain a competitive advantage to ensure the long-term development of the enterprise. In the process of international operation of enterprises, resources need to be allocated on a global scale [12]. Due to the existence of various uncertainties such as market prices and competitors, the operational risks faced by enterprises are getting higher and higher. Zhang et al. found that, in addition, with the expansion of enterprise scale, business expansion, the capital demand of enterprises is also increasing continuously; to raise funds with minimal capital cost in the international capital market, it is also related to the life and death of enterprises [13]. Hamzenejad et al. found that, after joining the WTO, China has further liberalized the foreign trade import and export rights of enterprises, Chinese products can be found in more than 100 member countries (regions) of the WTO, enjoying multilateral and stable most-favored-nation treatment, this makes the multinational business activities of Chinese enterprises more frequent and complex, therefore, it will inevitably lead to the multinational financial activities of enterprises, and it will involve a large amount of foreign exchange settlement business in multiple currencies, increasing foreign exchange transaction and translation risk. At the same time, with the further liberalization of current items and capital items, it will also further increase the risk of foreign exchange transactions and translation [14]. Zhang et al. found that, in addition, after joining the WTO, more and more foreign financial institutions will enter China, enterprises will have more financing channels and methods, the connection with the international capital market is closer, but at the same time, it also increases the exchange rate risk of enterprises, and it further enhances the impact of foreign exchange risk on enterprises [15].

## 3. Methods

Clustering algorithm is a type of unsupervised learning in the field of machine learning. In supervised learning, we have a labeled sample set as a training set beforehand; the samples are marked as two types *A* and *B*. A supervised model can find the boundaries of classes *A* and *B*; this classifier distinguishes the types of labels based on variable features. If the classifier is used to predict the test set data, we can use the prediction accuracy to evaluate the effect of the classification model. The K-means clustering algorithm is based on minimizing the square inner cluster sum (within a square cluster). The algorithm needs to specify the number of clusters, and the number of clusters is represented by the parameter *k*. The K-means clustering algorithm assigns the samples in the set to *K* disjoint clusters; each cluster has its own mean, called the “centroid.” By minimizing the within-cluster sum of squares, we can search for the most suitable centroids. The intracluster sum of squares is a distance measure that represents the degree of aggregation within a cluster. The larger the intracluster sum of squares, the smaller the average gap between the sample points in the cluster, and the easier it is for these sample points to be considered to belong to the same class [16, 17]. We use the function dist to represent a distance metric; then it satisfies the following four properties:

  Nonnegativity: dist(*x*_*i*_, *x*_*j*_) > 0.
  Identity: dist(*x*_*i*_, *x*_*j*_)=0.
  Symmetry: dist(*x*_*i*_, *x*_*j*_)=dist(*x*_*j*_, *x*_*i*_).
  Directness: dist(*x*_*i*_, *x*_*j*_) ≤ dist(*x*_*i*_, *x*_*k*_)+dist(*x*_*k*_, *x*_*j*_).

The K-means algorithm used uses Euclidean distance as a distance measure. Given samples *x*_*i*_=(*x*_*i*1_, *x*_*i*2_, *x*_*i*3_,…, *x*_*in*_) and *x*_*j*_=(*x*_*j*1_, *x*_*j*2_, *x*_*j*3_,…, *x*_*jn*_); then the definition of Euclidean distance is shown in formula (1):

(1) $$\begin{array}{c} E=\text{dist}\left(x_{i},x_{j}\right)=\sqrt{\sum_{u=1}^{n}{\left|x_{iu}-x_{ju}\right|}^{2}}. \end{array}$$

The larger the value of Euclidean distance, the farther the sample points of the cluster from the centroid, the smaller the similarity of the samples, and the worse the clustering effect; the smaller the value of Euclidean distance, the closer the sample points of the cluster to the centroid, the higher the similarity of the samples, and the better the clustering effect. The realization of K-means clustering algorithm is divided into three steps: In the first step, *K* samples are selected from the dataset as initial centroids; in the second step, each sample is assigned to the nearest centroid, forming a total of *K* clusters; in the third step, the mean of the samples in the cluster is used as the new centroid; in the fourth step, the second and third steps are repeated, and the position of the centroid is continuously updated until the iteration terminates. Since each iteration is smaller and gradually converged to the step distance of the centroid, the program typically presets the threshold to avoid too long operation times. The last position of the centroid is the basis for delineating clusters and determines which cluster belongs to each cluster. In order to demonstrate the learning process of the K-means algorithm, the author selected 2 financial indicators *X*_*i*_ and *Y*_*i*_ of 52 sample companies in a certain year; the learning process is as follows:

1. Establish a plane rectangular coordinate system, take the two financial indicators as the horizontal and vertical coordinates, and draw a scatter diagram of the sample enterprises.
2. Randomly select 2 samples as the initial centroids, which are represented by different colors.
3. According to the distance from the sample point to the two centroids, we can divide the samples into *A* and *B* types.
4. Calculate the average of the 2 financial indicators of Class $\bar{X_{A}}$ and Class $\bar{X_{B}}$ companies, get ${\bar{Y}}_{A}$ and ${\bar{Y}}_{B}$, and *C* and *D*, and use the result as the new centroid.
5. Repeat the process (3)∼(4) continuously, and the centroid will gradually move to the center of the respective groups.

**Figure 4:**
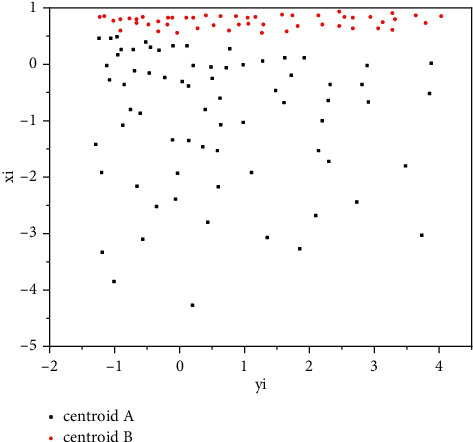
Schematic diagram of the results of the K-means clustering algorithm.

After the above process, we were able to divide the sample firms into Category *A* below and Category *B* above. As shown in Figure 4, *X*_*i*_ and *Y*_*i*_ are the standardized cash-to-assets ratio and current-liability ratio, respectively. After comparing the financial situation of the two types in the later period, we can find that the average financial performance of *B*-class companies is better than that of *A*-class companies [18, 19]. However, the K-means algorithm also has some shortcomings. Firstly, the premise of using Euclidean distance as an optimization criterion is that the clusters satisfy “convexity” and “isotropy.” If the sample data shows long bars or other irregular complex shapes in multidimensional space, then K-means clustering may not apply. Secondly, K-means clustering has the phenomenon of “curse of dimensionality.” Given the dimensions, the lower the value, the closer the distance; higher values indicate greater distances, but in the case of unknown dimensions, Euclidean distance also increases as the dimension increases. In addition, the results of the K-means clustering algorithm must converge in sufficient time, but the final returned result may only be a local optimum, because the result of clustering is highly dependent on the position of the initial centroid, so we need to select a different initial centroid each time, trial and error, until consistent results appear. Unlike the distance metric of the K-means clustering algorithm, Gaussian mixture clustering uses probability distribution as the criterion for clustering learning; its assumption is that all data are mixed from a finite number of multivariate Gaussian distributions, but the parameters are unknown. Before explaining Gaussian mixture clustering further, we need to define the multivariate Gaussian distribution. In the *n* dimension, there is a random vector *x* in the sample space and in the space, if the distribution of *x* is a Gaussian distribution, that is, a normal distribution, then its probability density function is as in formula (2).

(2) $$\begin{array}{c} p\left(x\right)=\frac{1}{\left(2\pi \right)n/2\left|\sum \right|1/2}e^{-1/2}{\left(x-\mu \right)}^{T}\sum^{-1}\left(x-\mu \right). \end{array}$$

The above is the basic principle of the Gaussian mixture model, and in the actual analysis, we can use the expectation-maximisation method to obtain the parameters of the model. First, the posterior probability is obtained through the initial parameters, then update the above three parameters, and perform multiple rounds of iterations, until the likelihood function LL(D) reaches the maximum value or changes less, and the final result returned is the Gaussian mixture model we need.

The evaluation standard of clustering effect mainly refers to the prediction of “special treatment” events by the model. A “special treatment” company is generally a company in financial distress; if we can find that a large number of “special treatment” companies fall into the same cluster, this cluster can then be called a set of high-risk companies. This involves the problem of assigning labels to clusters. In addition, the proportion of “specially treated” companies in the high-risk cluster also reflects the level of risk. Whether a company falls into the high-risk cluster, it can represent the true or false of the prediction results, and whether a company is a “special treatment” company, it can represent the true and false of the real situation. There are four situations in this dichotomous problem: true (true positive), the true example is predicted to be true; false positives, false positives are predicted to be true; for true negatives, false cases are predicted to be false; for false negatives, true cases are predicted to be false [20, 21].

According to Table 1, we can find that TP + FP + TN + FN is equal to the number of all samples, TP + FN is the number of all actual true samples, and TP + FP is the number of all predicted true samples. To evaluate predictive performance of models, we can introduce two indicators, Recall and Precision, whose definitions are shown in equations (3) and (4), respectively.

(3) $$\begin{array}{c} \text{Recall}=\frac{\text{TP}}{\text{TP}+\text{FN}}, \end{array}$$

(4) $$\begin{array}{c} \text{Precision}=\frac{\text{TP}}{\text{TP}+\text{FP}}. \end{array}$$

**Figure 5:**
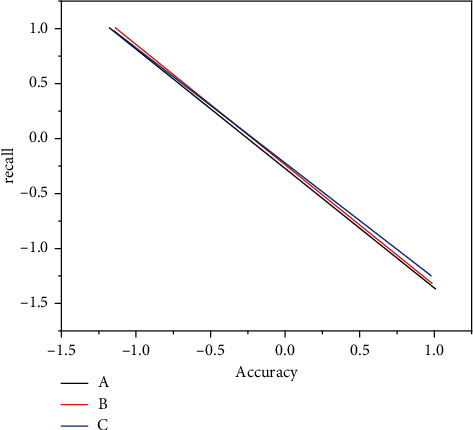
Schematic diagram of *P*-*R* curve.

Combined with the identification of financial risks, we use TP to denote the number of high-risk companies that are “specially treated,” use FN to denote the normal number of companies that are “specially treated,” and use FP to denote the number of normal companies that are not “specially treated.” Recall refers to the proportion of actually positive samples among the predicted positive samples and the accuracy rate represents the ratio of samples in the cluster of true samples in the cluster. Ideally, if all true samples are predicted to be true and at the same time all fake samples are predicted to be fake, then the recall and precision are both 100%. The larger the cluster size is, the more the samples are expected to be true, with the actual sample being included, and the recall rate increases. However, if the cluster size increases, there are many false samples contained in the cluster, so the precision is reduced. Therefore recall and precision are contradictory metric pairs. Suppose we have obtained a classifier by training, and arrange the samples in descending order according to the probability of being true, and use the classifier to predict from top to bottom, and gradually expand the scale of the sample; the recall rate and precision rate will also gradually change. If we draw these two indicators in two-dimensional coordinates, we can get a quasi-convex curve, that is, the *P*-*R* curve.  Figure 5 shows two curves *A* and *B*; we can know that the *B* curves in the figure wrap a curve. If the recall rate is given, the precision rate of the *B* curves in the figure wraps a curve; if the precision rate is given, the recall rate of the *B* curve is higher than that of the A curve. We can conclude that the *B* curve model always outperforms A [22, 23].

But if one curve cannot wrap another curve (such as curve *A* and curve *C*), then, we cannot directly judge the quality of the model through the recall rate and precision rate. In this case, we can usually introduce a composite indicator. As shown in Figure 4, the dashed line from the origin intersects the curve, the position of the intersection point shown by the arrow is the balance point that comprehensively considers the recall rate and the accuracy rate, and this point can be used to judge whether the model is good or bad. Based on this idea, we introduce the commonly used F1 score indicator below, that is, the harmonic average of the two.

(5) $$\begin{array}{c} \text{F}1=\frac{2\times \text{Precision}\times \text{Recall}}{\text{Precision}\times \text{Recall}}=\frac{2\times \text{TP}}{2\text{ TP}+\text{FP}+\text{FN}}. \end{array}$$

Its general form is shown in formula (6).

(6) $$\begin{array}{c} F_{\beta }=\frac{\left(1+\beta^{2}\right)\times \text{Precision}\times \text{Recall}}{\left(\beta^{2}\times \text{Precision}\right)+\text{Recall}}. \end{array}$$

**Figure 6:**
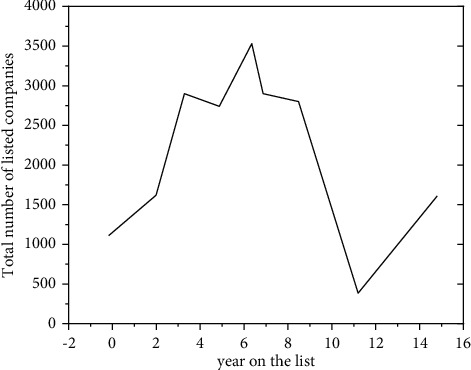
The total number of listed companies from 1997 to 2018 and the number of companies listed on ST in the following year.

“Special treatment” companies are different from companies that are normally listed and companies that have terminated their listing. At the beginning of the implementation of the “special treatment” system, the regulators have made a good screening of corporate risks based on the financial status of listed companies. As shown in Figure 6, since the implementation of the “special treatment” system in 1998, until 2006, the proportion of “special treatment” companies in the total number of A-share listed companies has steadily increased. In 1997, there are 700 listed companies in China's A-share market; of these, 25 were due to deteriorating financial conditions or other reasons; in the second year, it was listed as a “special treatment” type of enterprise, 4% of the total number of listed companies. In 2006, the proportion of “special handling” companies rose to 10%, the highest in history.

But, due to the incomplete exit mechanism of Chinese listed companies, the actual warning effect of the “special handling” system is not as effective as one might expect. As can be seen from Figure 6, after 2008, with the increase in the number of listed companies, the number and proportion of “special treatment” companies both declined and were maintained at a low level; there are many reasons for this phenomenon. On the one hand, the threshold for initial public offering of domestic listed companies is relatively high, companies that can meet the listing conditions are usually stable companies in the mature or mid-development stage, operating conditions are good, and most companies can resolve financial crises in a timely manner. On the other hand, as to whether the listed company should terminate the listing, although the regulator will determine it according to certain standards, the reference indicators are not comprehensive enough. Listed companies can legally beautify their financial reports through various means, so as to protect yourself from hitting the red line of terminating the listing.

## 4. Experiments and Analysis

**Figure 7:**
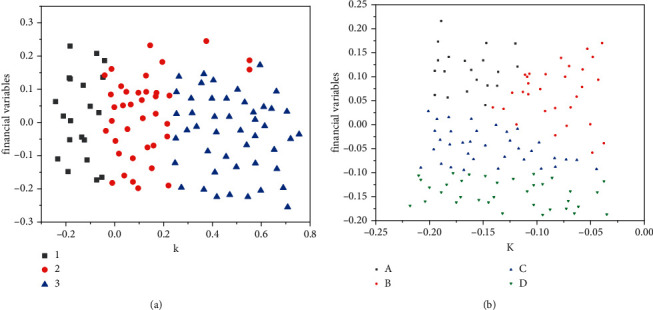
Schematic diagram of K-means clustering after PCA dimensionality reduction.

The PCA algorithm and the K-means clustering algorithm are from the Scikit-learn toolkit in the Python programming software; the training of the model is divided into three steps: The first step, using principal component analysis, reduce the 4 financial variables of all samples to 2 dimensions; the second step is to perform the first K-means clustering (*K* = 3) and divide all samples into 3 clusters, as shown in the left image of Figure 7; the third step is to perform K-means clustering again (*K* = 4) with the cluster containing the most “specially treated” companies, as shown in the right image of Figure 7; in the fourth step, again we get the cluster with the most “special treatment” companies and label it as a high-risk cluster [24, 25].

After two steps of K-means clustering training, we get the clustering result, some of the results are shown in Table 2, and the centroids with an asterisk “^*∗*^” in the table are the centroids of high-risk clusters.

According to the training results, we identified the cluster with the most new “special treatment” companies as the high-risk cluster, all companies in the cluster are so-called “high-risk companies.” The high-risk cluster includes both companies that were “specially treated” in the following year; it also includes those who have not been judged as “special treatment,” but businesses with financial status similar to “special treatment” companies. If the year in which the listed company publishes its financial report is set as year *Y* (annual financial report should be published before the end of April of the following year), then as of *Y* + *N* years, if a new “special treatment” company emerges in the high-risk cluster, then this classification warning model is effective; the proportion of “specially treated” companies can also indicate how effective the clustering model is. In addition, the probability of a “high-risk company” being placed on the “special treatment” list can be expressed using recall and precision.

Table 3 shows the identification of financial risks by the K-means clustering algorithm. The year in the first row of the table is 2008; the company released its 2007 financial statements that year. In this year, the high-risk cluster contains 118 companies, accounting for 8% of the total number of listed companies, but this cluster was able to contain 36.0% of the new “special treatment” companies. As of year *Y* + 1, 2009, 10.2% of the companies in the high-risk cluster entered the “special treatment” list. As of *Y* + 2 years, this ratio rose to 17.0%, and as of *Y* + 3, twenty percent of companies in the high-risk cluster will be identified as “specially treated” companies. As can be seen from Table 3, except for 2014 and 2015, the clusters formed by the K-means clustering model are not large in scale, accounting for 5.5% to 7.9% of the total number of listed companies, but these small clusters contain many new “special treatment” companies. According to the statistics, the proportion of newly added “specially treated” companies in the total number of listed companies is less than 2.5% every year, and it is kept within 2% all year round; the average accuracy of high-risk clusters in predicting risk warning events in the next year is 9.6%. From this it can be seen that, with clustering high-risk clusters using K-means, the identification of financial risks of listed companies has certain effects [26].

## 5. Conclusion

What is discussed is the role of K-means clustering algorithm in the identification of financial risks of listed companies. First, with a metric based on Euclidean distance, we can discover commonalities in financial data of “specially treated” companies and filter financial indicators for K-means clustering algorithm; thereby, the input variables of the model are determined. From 27 alternative financial indicators, finally, 4 financial variables (tangible assets ratio, cash assets ratio, current debt ratio, and noncurrent debt ratio) were selected. Contrary to previous literature conclusions, after using Euclidean distance to rank the clustering effect of “specially treated” companies, it is found that the first few financial indicators that make the most close clusters are neither common profit indicators, such as net profit margin, return on equity, and return on assets, nor a growth indicator that reflects the long-term development of the company, such as the growth rate of fixed assets, etc., but liquidity indicators. After dimensionality reduction using principal component analysis, we performed K-means clustering twice; finally, the cluster containing the most new “special treatment” companies was designated as the high-risk cluster. Turn out: although the number of high-risk companies selected by the K-means algorithm is small, only 9% of the full sample, the high-risk cluster can contain nearly 30% of the new “special treatment” companies. If the time period is extended to the next five years, this proportion will be even higher. If the prediction of “special handling” events is used as the criterion for evaluating high-risk clusters, then K-means clustering can effectively screen out those risky companies that need to be treated with caution by investors.

## Figures and Tables

**Table 1:** Prediction result matrix.

Forecast situation	The true situation	
	Real	Fake
Just	TP (true)	FP (false positive)
Opposite	FN (false negative)	TN (true negative)

**Table 2:** Partial K-means clustering results.

Fiscal year	Centroid location	
2007	(−0.17, −0.03) (−0.17, 0.09)^*∗*^	(−0.17, −0.15) (−0.09, 0.06)
2010	(−0.16, −0.07) (0.01, 0.01)	(0.05, −0.12) (−0.12, −0.20)^*∗*^
2013	(−0.20, 0.00) (−0.16, −0.11)^*∗*^	(−0.11, 0.12) (−0.24, 0.15)
2016	(−0.10, −0.03) (−0.16, 0.10)	(−0.17, 0.35) (−0.21, −0.04)^*∗*^
2018	(−0.21, −0.04)^*∗*^ (−0.15, 0.33)	(−0.10, −0.03) (−0.15, 0.11)

**Table 3:** The screening effect of K-means clustering on financial risk.

years	High-risk cluster proportion (%)	Number of high-risk companies (homes)	*Y* + 0 year recall rate (%)	*Y* + 1 year accuracy rate (%)	*Y* + 2 year accuracy rate (%)	*Y* + 3 year accuracy rate (%)	*Y* + 4 year precision rate (%)
2008	8	1 18	36.0	10.2	17.0	20.3	20.3
2009	2.4	38	17.2	23. I	23	23	23、
2010	6.2	105	20.5	10.5	14.3	15.2	16.2
2011	6.0	123	31.3	7.2	8.9	11.4	16.3
2012	5.5	126	19.2	6.4	7.9	9.5	12.0
2013	6.5	157	22.7	8.3	10.8	13.4	16.6
2014	21.0	515	55.6	6.6	9.7	12.4	16
2015	16.4	784	20.5	4.3	7.1	10.0	12.6
Mean value	9.0	246	27.9	9.6	12.4	14.4	16.6

## Data Availability

The labeled dataset used to support the findings of this study is available from the corresponding author upon request.
